# The genome sequence and effector complement of the flax rust pathogen *Melampsora lini*

**DOI:** 10.3389/fpls.2014.00098

**Published:** 2014-03-24

**Authors:** Adnane Nemri, Diane G. O. Saunders, Claire Anderson, Narayana M. Upadhyaya, Joe Win, Gregory J. Lawrence, David A. Jones, Sophien Kamoun, Jeffrey G. Ellis, Peter N. Dodds

**Affiliations:** ^1^CSIRO Plant IndustryCanberra, ACT, Australia; ^2^The Sainsbury Laboratory, Norwich Research ParkNorwich, UK; ^3^Research School of Biological Sciences, College of Medicine, Biology and Environment, Australian National UniversityCanberra, ACT, Australia

**Keywords:** rust, flax, *Melampsor*a, effector, virulence, avirulence

## Abstract

Rust fungi cause serious yield reductions on crops, including wheat, barley, soybean, coffee, and represent real threats to global food security. Of these fungi, the flax rust pathogen *Melampsora lini* has been developed most extensively over the past 80 years as a model to understand the molecular mechanisms that underpin pathogenesis. During infection, *M. lini* secretes virulence effectors to promote disease. The number of these effectors, their function and their degree of conservation across rust fungal species is unknown. To assess this, we sequenced and assembled *de novo* the genome of *M. lini* isolate CH5 into 21,130 scaffolds spanning 189 Mbp (scaffold N50 of 31 kbp). Global analysis of the DNA sequence revealed that repetitive elements, primarily retrotransposons, make up at least 45% of the genome. Using *ab initio* predictions, transcriptome data and homology searches, we identified 16,271 putative protein-coding genes. An analysis pipeline was then implemented to predict the effector complement of *M. lini* and compare it to that of the poplar rust, wheat stem rust and wheat stripe rust pathogens to identify conserved and species-specific effector candidates. Previous knowledge of four cloned *M. lini* avirulence effector proteins and two basidiomycete effectors was used to optimize parameters of the effector prediction pipeline. Markov clustering based on sequence similarity was performed to group effector candidates from all four rust pathogens. Clusters containing at least one member from *M. lini* were further analyzed and prioritized based on features including expression in isolated haustoria and infected leaf tissue and conservation across rust species. Herein, we describe 200 of 940 clusters that ranked highest on our priority list, representing 725 flax rust candidate effectors. Our findings on this important model rust species provide insight into how effectors of rust fungi are conserved across species and how they may act to promote infection on their hosts.

## Introduction

Ever since the work of Flor (1955), the interaction between the flax plant (linseed; *Linum usitatissimum*) and the flax rust fungus *Melampsora lini* (*Mli*) has served as a model pathosystem to study the genetics underlying host-pathogen interactions in plants. Flor's work led to the formulation of the gene-for-gene model describing the interaction between host resistance (R) genes and pathogen avirulence (Avr) genes (Flor, 1955). This gene-for-gene relationship was later found to apply to most interactions between plants and their adapted pathogens and pests, both in natural, or agricultural systems (Jones and Dangl, 2006). In the 1990s, rust resistance genes from flax were among the first R genes to be cloned in plants, followed by the identification of flax rust Avr genes as encoding secreted proteins that activate R gene-encoded intracellular immune receptors containing Toll-like nucleotide-binding leucine-rich repeats (TIR-NB-LRR) domains (reviewed in Ellis et al., 2007). To date the flax rust pathosystem continues to play an important role in the genetic dissection of plant-pathogen interactions (Lawrence et al., 2007; Ravensdale et al., 2010). For example, the feasibility of genetic transformation and artificial gene silencing in flax rust (Lawrence et al., 2010) makes it an important pathosystem for the study of virulence in biotrophic fungi. However, to date genomic resources have been lacking. In this study, we remedy this by describing the genome sequence of the flax rust fungus.

Rust fungi (Phylum Basidiomycota, order Pucciniales) constitute the largest group of fungal pathogens of plants, with more than 7000 species described (Cummins and Hiratsuka, 2003). They are responsible for considerable yield losses in cultivated crops such as wheat or barley, with wheat stem rust alone having the potential to cause global losses of up to USD 54 billion per annum (Pardey et al., 2013). They can also significantly impact biodiversity, e.g., myrtle rust (*Uredo rangelii*) is a recently introduced rust species in Australia currently spreading on Myrtaceae on a continental scale threatening many native tree species and ecosystems (Australian Nursery and Garden Industry, 2012). Consequently, understanding mechanisms of virulence in rust fungi and devising innovative ways to protect crops against them is essential.

Rust fungi are obligate biotrophs, that is they need one or more living hosts to grow and complete their complex reproductive cycle. During infection they form haustoria, specialized structures surrounded by invaginated host cell membrane, with a role in nutrient uptake from the host and delivery of secreted effector proteins into host cells (Mendgen and Nass, 1988; Kemen et al., 2005; Rafiqi et al., 2010). These effectors are proposed to promote pathogen reproductive fitness by mediating the suppression of host immunity, creating a suitable environment for the pathogen and promoting nutrient uptake (see review by Duplessis et al., 2011c). For example, the RTP1p effector, originally identified in the bean rust pathogen, is delivered into host cells during infection and may act as an inhibitor of host proteases to promote disease (Kemen et al., 2005; Pretsch et al., 2013). Importantly, a subset of these effectors elicit host resistance, including four Avr genes cloned from *M. lini* (*AvrL567*, *AvrM*, *AvrP123*, and *AvrP4*; Dodds et al., 2004; Catanzariti et al., 2006; Barrett et al., 2009), and one recently identified Avr candidate from the wheat stem rust fungus (Upadhyaya et al., 2013). Their function in pathogenicity is unknown. A further eight Avr loci from *M. lini* have been genetically characterized, as well as one inhibitor gene that specifically suppresses host resistance against normally avirulent isolates (Lawrence et al., 1981; Jones, 1988; Lawrence, 1995). Consistent with their role in mediating adaptation of rust fungi to their hosts, rust genes encoding effectors can exhibit high levels of polymorphism and signatures of positive selection (Dodds et al., 2006; Van Der Merwe et al., 2009; Joly et al., 2010). Thus, identifying the effectors possessed by rust fungi to infer their function and the evolutionary processes acting on them is key to understanding mechanisms of pathogenicity in rust fungi and the evolution of their often narrow range of host species. The elucidation and comparisons of the genome sequences of rust pathogen species is an important step toward achieving this goal (McDowell, 2011).

Over the past few years, a number of rust fungi genomes have been sequenced, including the poplar leaf rust pathogen *Melampsora larici-populina* (*Mlp*, ~101 Mbp), a close relative of *Mli*, as well as the wheat and barley stem rust pathogen *Puccinia graminis* f.sp. *tritici* (*Pgt*, ~88 Mbp; Duplessis et al., 2011a) and the wheat yellow (stripe) rust pathogen *P. striiformis* f.sp. *tritici* (*Pst*, between 65 and 130 Mbp; Cantu et al., 2011; Zheng et al., 2013). In comparison, the dikaryotic genome of *M. lini* uredospores (2n = 36; Boehm and Bushnell, 1992) was estimated from nuclear fluorescence studies to be ~2.5 times larger than that of *Pgt*, giving a predicted size of ~220 Mbp (Eilam et al., 1992). In addition, transcriptome analyses have identified rust fungi genes expressed during infection, including effectors, in flax rust (Catanzariti et al., 2006), poplar rust (Duplessis et al., 2011b; Hacquard et al., 2012), wheat stripe rust (Yin et al., 2009; Cantu et al., 2013; Garnica et al., 2013), faba bean, common bean and soybean rusts (*Uromyces viciae-fabae*, *U. appendiculatus* and *Phakopsora pachyrhizi*, respectively; Jakupović et al., 2006; Link and Voegele, 2008; Link et al., 2013) and coffee rust pathogens (Fernandez et al., 2011). The availability of genome and transcriptome sequences from multiple rust species and isolates allows interspecies comparisons to identify shared rust fungal effectors and determinants of host specificity both among and within species (Duplessis et al., 2011a; Saunders et al., 2012; Cantu et al., 2013).

The total number of effectors in the flax rust fungus and how many are unique to this species is unknown. To gain insights, we sequenced and annotated the genome of *Mli* isolate CH5. Taking advantage of previous knowledge of flax rust avirulence genes, we then characterized its predicted effector complement in relation to those of three other rust species. The availability of a sequenced genome and a compilation of candidate effectors from the flax rust fungus together with the available genetic tools, will help in future studies to identify determinants of host specificity in the flax-flax rust interaction as well as better understanding the mechanisms of rust pathogen infection.

## Results

### *de novo* genome assembly and annotation

We selected the flax rust pathogen isolate CH5, the F1 parent of a well-characterized F2 family segregating for 10 Avr and one inhibitor loci (Lawrence et al., 1981) to build the *Mli* reference genome sequence. Illumina sequencing data were obtained using paired-end and mate-paired libraries of four sizes (~300, 2000, 3000, and 5000 bp; Table S1). The genome assembly and initial scaffolding of contigs were performed using *SOAPdenovo* with a k-mer value of 41, followed by multiple rounds of gap-closing and scaffolding. The final 189 Mbp genome assembly (including 14.1% of *N*'s) consisted of 21,310 scaffolds and represented 86.4% of the predicted 220 Mbp genome size (Table 1). A *de novo* search for repetitive elements identified ~45% of the genome sequence as interspersed repeats (Table 2).

**Table 1:** **Summary statistics of assembly and annotation of the genome of flax rust pathogen *Melampsora lini* isolate CH5**.

Cumulative size of scaffolds	189.5 Mbp (86.4% of expected size)
No. scaffolds	21,310
Fraction of *N*'s in assembly	14.1%
Longest scaffold	239.7 kbp
N50 scaffold length	31.5 kbp
L50 scaffold count	1799
GC content	41%
Gene space completeness (CEGMA)	95%
Protein-coding genes	16,271
Mean scaffold size	8.9 kbp
Median scaffold size	1.1 kbp
No. scaffolds >1 Mbp	0
No. scaffolds >100 kbp	81 (0.4% of scaffolds)
No. scaffolds >10 kbp	5339 (25.1% of scaffolds)
No. scaffolds >1 kbp	10,798 (50.7% of scaffolds)

Only scaffolds larger than 200 bp were retained in the final assembly. The N50 of scaffold length indicates that 50% of the total assembled sequence is on scaffolds larger than that size. The L50 scaffold count indicates the number of scaffolds larger than the N50 length. Gene space completeness indicates the fraction of 248 conserved eukaryotic genes (CEGMA) present with >70% length in the assembly.

**Table 2:** ***De novo* identification of sequence repeats in the genome of the flax rust pathogen *Melampsora lini* isolate CH5**.

**Type of repeat**	**Number of elements**	**length (kbp)**	**Percentage occupied of sequence**
**INTERSPERSED REPEATS**			
Retrotransposons			
LTR elements	84,252	42,907	22.64
LINEs	3641	2834	1.5
incl. LINE1	179	133	0.07
incl. LINE2	320	188	0.1
incl. L3/CR1	155	31	0.02
SINEs of type MIRs	70	45	0.02
DNA transposons	37,834	14,273	7.53
Unclassified	81,255	25,603	13.51
**OTHER**			
Simple repeats	19,637	1114	0.59
Low complexity	4212	237	0.13
Satellites	190	63	0.03
Small RNA	129	58	0.03
Total detected		87,008	45.91%

Retrotransposons include LTRs, long tandem repeats; LINEs, long interspersed elements; and SINEs, short interspersed elements of sub-class; MIRs, mammalian interspersed repeats.

**Figure 1:**
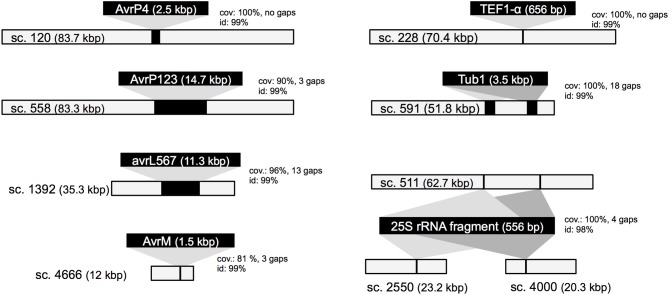
**Validation of the *M. lini* isolate CH5 genome assembly**. Seven previously sequenced genomic loci were used as controls (black bars with text, not in scale). They were aligned to scaffolds from the *de novo* assembly. Scaffolds showing the best match are shown (light gray bars, in scale), with alignment scores including portion of query covered in match (cov.), sequence identity (id.), and alignment gaps (in bp).

To assess the gene space coverage in the genome assembly we used three different sources of evidence. First, an analysis searching for the CEGMA set of 248 conserved eukaryotic genes (Parra et al., 2009) in the assembly found 95% of them present “in full,” indicating a high level of completeness for the genome assembly. In a second test, we used EST sequences from an haustorial cDNA library from Catanzariti et al. (2006). In addition to the 856 ESTs previously described we sequenced an additional 1937 cDNA clones. After filtering out ESTs coming from flax, flax rust ribosomal RNA or retrotransposons, of the 1399 remaining ESTs, only 3 (0.2%) did not match the assembled *Mli* genome but did match genes of other fungal species including *Mlp*, *Melampsora magnusiana*, and *Magnaporthe oryzae*, again supporting that most of the gene space is covered in the assembled genome sequence. Finally, the assembly was checked against a total of 79 kbp of genomic sequences from *Mli* previously derived by Sanger sequencing of cloned DNA. The sequenced regions serving as positive controls included loci carrying *AvrP123*, *AvrP4*, *AvrL567-C*, *AvrM-A*, *AvrM-B*, *AvrM-C*, *β-tubulin*, *transcription elongation factor 1α* and a gene fragment from 25S ribosomal RNA. All tested regions were present in full at least once in the assembly, with the exception of regions containing genes from the *AvrM* family (Figure 1). In that case, the five previously sequenced paralogs *AvrM-A*, *-B -C, -D*, and *-E* from the avirulence haplotype and *avrM* from the virulence allele in isolate CH5 were assembled as a single gene sequence corresponding to the coding region of *AvrM*, whereas their repeat-rich flanking regions were assembled as separate contigs. Such “collapse” of paralogs into a single assembled sequence was not seen in all cases, e.g., two paralogs of the *β-tubulin* (TUB1) gene were found on the same scaffold, consistent with expectations (Ayliffe et al., 2001), and four copies of 25S rRNA fragment were assembled, including two on the same scaffold (Figure 1). Altogether, this suggests that the *Mli* gene space, including that of complex gene families and effectors, is mostly present in the assembly. Also, we have found that, on the limited number of genomic regions tested, the sequence contiguous to genes has been assembled mostly correctly.

**Figure 2:**
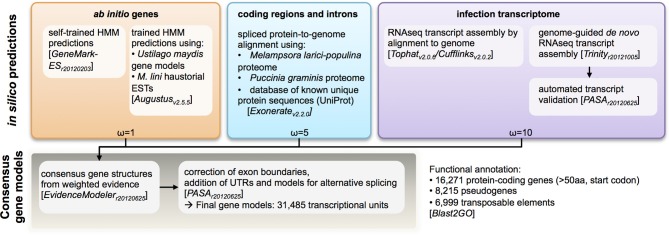
**Analysis pipeline for annotation of protein-coding genes in the genome of the flax rust pathogen *Melampsora lini.*** Multiple types of evidence were assigned weights (ω) and aggregated to generate a consensus call for a transcriptional unit.

To aid gene annotation, we generated a transcript assembly based on RNAseq data (~58 million reads, 75 bp single-end) from an RNA sample collected from rust infected flax leaves 6 days post infection. The transcriptome assembly was performed using both assembly-by-alignment in *Cufflinks* and genome-guided *de novo* assembly in *Trinity*. To identify protein-coding genes in the assembled genome scaffolds, several types of evidence were weighted and aggregated to derive consensus gene calls (Figure 2). In order of decreasing weights (ω in Figure 2), this evidence included: (1) assembled transcripts from the RNAseq library and haustorial-specific ESTs; (2) spliced protein-to-genome alignments using *Mlp* and *Pgt* proteomes; and (3) *ab initio* gene predictions. In total, 31,485 transcriptional units were identified, including 6999 derived from predicted transposable elements, 8215 pseudo-genes (predicted proteins less than 50 amino-acids long or missing a start codon) and 16,271 protein-coding genes. This is similar to the 16,399 genes identified in *Mlp* (Duplessis et al., 2011a). To validate the annotation process, we found that 98% of the conserved eukaryotic (CEGMA) genes assembled “in full” (95% of total) were correctly annotated as protein-coding genes with correct protein sequence. Therefore, the *Mli* assembly presented herein likely contains most of the gene space, with largely complete protein sequences. Version 1 of the genome and proteome sequence and annotation can be found in Additional file 1 (NCBI Bioproject ID PRJNA239538). Also, the current genome sequence and annotation can be browsed at and downloaded from http://webapollo.bioinformatics.csiro.au:8080/melampsora_lini.

### Genomic evidence for nutrient assimilation pathways in the flax rust fungus

Based on genome comparisons with non-biotrophic basidiomycetes, it has been hypothesized that the evolution of obligate biotrophy in rust pathogens is associated with reductions in metabolic abilities via losses of whole metabolic pathways, coupled with an expansion in transporter gene families for enhanced uptake of host-derived nutrients. For example, previous studies noted the absence of some members of the nitrate assimilation cluster in several rust fungi (Duplessis et al., 2011a; Garnica et al., 2013). Within the *Mli* genome assembly, we identified a putative nitrate reductase gene (MELLI_sc3720.2) adjacent to a Major Facilitator Superfamily (MFS) transporter (MELLI_sc3720.1) of unknown function, which may correspond to the nitrate/nitrite transporter in the cluster. However, we did not identify a gene encoding a nitrite reductase and the nitrate reductase gene appeared to be expressed at an extremely low level in infected tissue, suggesting that this pathway may not be functional in *Mli*, similar to other rust fungi. On the other hand, homologs of all components of the ammonium assimilation pathway were identified in the *Mli* genome, including four ammonium transporters (MELLI_ sc457.12 MELLI_sc152.7 MELLI_sc152.8), the key enzymes glutamate synthase (MELLI_sc11.10_sc11.11) and glutamine synthetase (MELLI_sc3079.2), NAD-specific glutamate dehydrogenase (MELLI_sc1197.2), aspartate aminotransferases (MELLI_sc30.24 and MELLI_sc1978.2), asparagine synthase (MELLI_sc1344.1 and MELLI_sc1683.3) and asparaginase (MELLI_sc2460.3). Thus, as proposed for *Mlp*, the major uptake of host-derived nitrogen is likely in the form of ammonium. In addition, *Mli* may also acquire amino acids and carbon via a relatively large number of amino acid and peptide transporters. The genome of *Mli* contained 16 amino acid permeases/transporters including homologs of *Uromyces vicie-fabae* AAT1, AAT2 and AAT3 (MELLI_sc114.5, MELLI_sc1561.1, and MELLI_sc1251.10, respectively) and 27 putative oligopeptide transporters, which is slightly above the 22 detected in *Mlp*. Several components of the sulfate assimilation pathway were identified in *Mli*, including four sulfate transporter genes (MELLI_sc1698.3, MELLI_sc2898.2, MELLI_sc487.5, and MELLI_sc610.2), sulfite reductase α and β subunits (MELLI_sc3167.3 and MELLI_sc1053.1, respectively) and phosphoadenosine phosphosulfate reductase (MELLI_sc358.2), although we did not identify an ATP sulfurylase. Similar observations were made for *Mlp* (Duplessis et al., 2011a). In *Mli*, expression levels in infected tissues varied greatly among the putative members of the pathway, with one putative transporter (MELLI_sc487.5) and the sulfite reductase α subunit showing no expression and the phosphoadenosine phosphosulfate expressed at very low level. Other genes identified as putative components of the pathway were all highly expressed, suggesting an important role for them during infection. It is thus unclear whether the seemingly almost complete *Mli* sulfate assimilation pathway is functional in its entirety. Overall, this suggests that the two *Melampsora* species studied here have largely similar metabolic abilities.

In total, we have identified 190 putative proteins showing similarity to transporters from other species (Additional file 2, “Transporters”). There were at least 65 members of the Major Facilitator Superfamily (MFS), with 13 putative sugar transporters. This included homologs of the hexose transporter *Uvf*HXT1 and its associated H+/ATPase transporter *Uvf*PMA1 (MELLI_sc2238.1 and MELLI_sc2503.1, respectively). In addition, 17 ATP-binding cassette (ABC) transporters were identified. This was less than the numbers detected in *Mlp* and *Pgt* for both MFS transporters (88 and 51, respectively) and ABC transporters (50 and 38, respectively) (Duplessis et al., 2011a). We also identified two putative auxin efflux carriers (MELLI_sc2698.1 and MELLI_sc890.3), compared with 7 identified in *Mlp*. Fungal pathogens can synthesize auxin, which may be secreted into the host during infection to promote disease (see review by Wang and Fu, 2011). Overall, the 190 putative transporters identified in *Mli* is significantly less than the 356 identified in *Mlp*, although this reduction was not uniform across the different kinds of transporters, e.g., for oligopeptide transporters (see above) we found a larger number in *Mli* than *Mlp*.

### Comparative study of effector complements from four rust fungi

#### Mining for candidate effectors in four species of rust fungi

**Figure 3:**
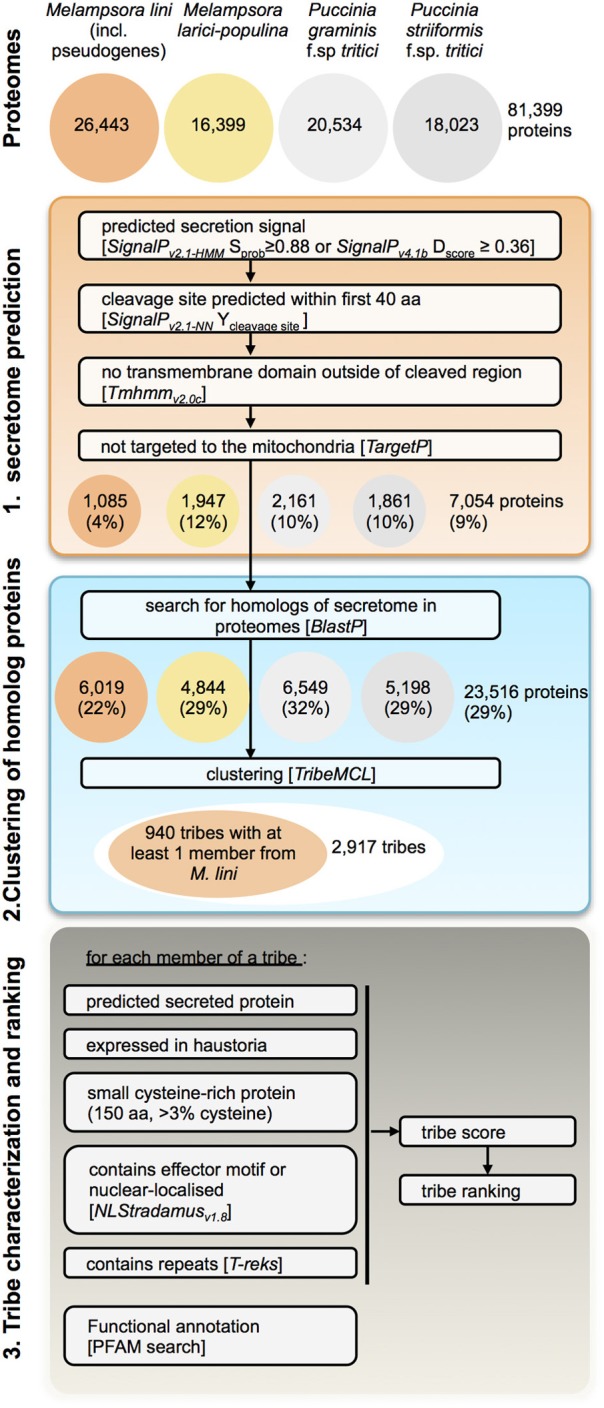
**Pipeline for prediction of candidate rust fungal effectors**. Tribes of predicted secreted proteins were gathered from the proteomes of *M. lini* isolate *CH5, M. larici-populina* isolate 98AG31, *P. graminis* f.sp *tritici* isolate CDL 75–36-700–3 (race SCCL) and *P. striiformis* f.sp. *tritici* isolate 130. Tribes containing at least one member from *M. lini* were selected for characterization and ranking.

To identify putative effectors within *Mli* and highlight those conserved across rust fungi species, we modified the prediction pipeline described in Saunders et al. (2012) to search the proteomes of four rust species, *Mli*, *Mlp*, *Pgt*, and *Pst* (Figure 3). Here we broadly define effectors as any fungal protein that is secreted by the fungal cell to act on host-derived substrates or targets or otherwise affect host responses. First, secretome predictions were performed by identifying proteins with a predicted signal peptide (SP), no transmembrane domain and no mitochondria-targeting motif, as described in Torto et al. (2003). To set the stringency of the selection criteria in the pipeline, we used known rust fungal effectors, including AvrL567, AvrM, AvrP123, AvrP4, RTP1p and their homologs from the four investigated rust pathogen species where relevant. The most stringent criteria that still allowed all the known rust fungal effectors to pass were used, e.g., a D-score value of 0.36 in *SignalP*_*v*4.1_. In total, 7054 (9%) of the 81,399 proteins from all four rust fungi were predicted to be secreted, including 1085 from the flax rust fungus. Subsequently, similarity searches were undertaken between the predicted secreted proteins and the remaining proteomes, to ensure candidates with mis-annotated N-termini or missed SPs were not overlooked. This resulted in 23,516 proteins being selected (29% of the total proteomes). Similarity-based Markov clustering grouped these into 2917 “effector tribes” (Additional file 2, “Complete list of tribes”), of which 940 tribes (16,908 proteins) contained at least one protein from the flax rust fungus and were used for further analysis.

**Figure 4:**
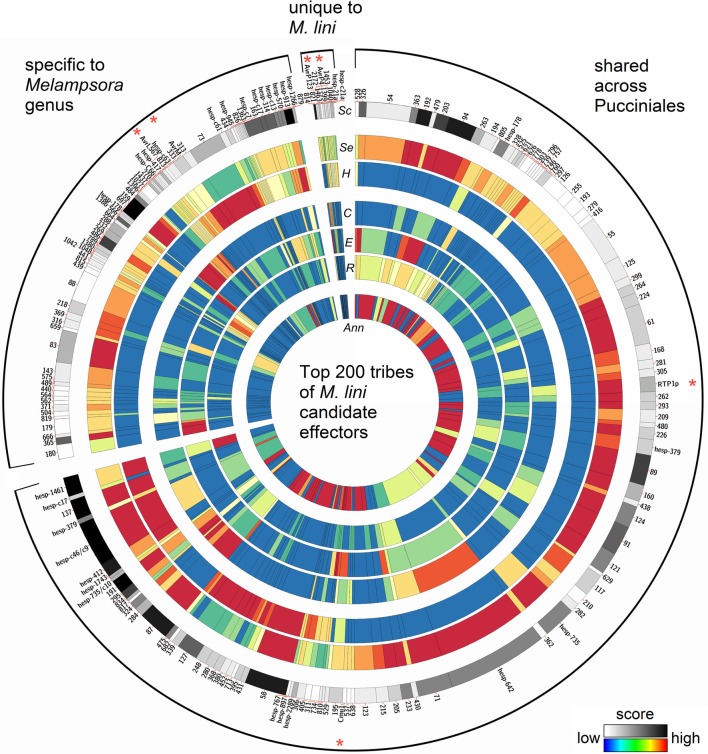
**Properties of the top 200 tribes of *M. lini* candidate effectors**. Tribes were assembled with 2642 proteins coming from four rust species (*Mli*, *Mlp*, *Pgt*, and *Pst*), and all contain at least one member from *Mli*. Tribes were grouped according to whether they contain members from *Mli* only or members from the two *Melampsora* species only or members from all four rust species. On a circle, each bar represents a tribe, with the width of bar proportional to the number of members in that tribe. Previously known *Mli* avirulence proteins and fungal virulence effectors are indicated with a red asterisk. For each tribe, heatmaps indicate the scores for (*Sc*) overall tribe rank, (*Se*) prediction of secretion, (*H*) similarity to haustorial ESTs, (*C*) presence of small cysteine-rich proteins, (*E*) presence of an effector motif or nuclear localization signal, (*R*) presence of tandem repeats, and (*Ann*) fraction of proteins with a PFAM annotation. Note that the (*H*) field represents similarity across the whole tribe with *Mli* haustorial ESTs, as opposed to haustorial expression of tribe members from all four rusts. As *Mli* is the focus of this study, tribes with members expressed in the haustoria of *Mli* were highlighted. Some previously known haustorially secreted proteins either from *Mli* or other rusts may thus appear to have low score.

These 940 tribes were characterized *in silico* for properties associated with known effectors from *Mli* and other filamentous plant pathogens. We considered tribes with a high fraction of: (1) predicted secreted proteins; (2) proteins with similarity to *Mli* haustorial ESTs or haustorial predicted secreted proteins (HESPs; Catanzariti et al., 2006); (3) small cysteine-rich proteins; or (4) proteins with predicted effector motifs such as [Y/F/W]xC (Godfrey et al., 2010) as high priority for further analysis. In addition, the presence of a nuclear localization signal or internal repeats in members of a tribe was considered. A single tribe score was then assigned for each of the 940 tribes to order them based on their probability of containing effector proteins (Saunders et al., 2012; Additional file 2, “Ranked tribes incl. *Mli* members”). To validate our approach, we looked at the ranking of tribes containing AvrP4, AvrP123, AvrM, AvrL567, RTP1p and rust homologs of the corn smut pathogen (*Ustilago maydis*) effector Chorismate mutase 1 (Cmu1; Djamei et al., 2011), which all occurred among the top 232 tribes out of 940 (Figure 4; Additional file 2, “Top 200 tribes PFAM annotation”). Manual inspection and curation resulted in removal of 32 clusters that did not appear to represent true effector candidates, mostly large clusters containing only one or a few predicted secreted proteins that may have been mis-annotated. The remaining top 200 tribes were selected for further analysis (Figure 4). Also ranking within the top 200 were tribes containing previously identified HESPs from *Mli* and PST130 homologs of HESPs from the stripe rust pathogen isolate 79 (Garnica et al., 2013).

#### The majority of candidate effectors show conservation across rust fungi

**Figure 5:**
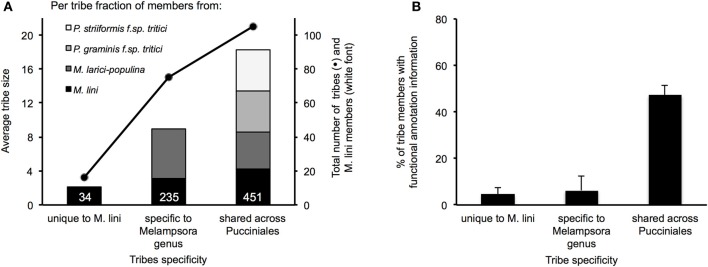
**Characteristics of the top 200 tribes of *M. lini* candidate effectors**. Tribes were assembled with 2642 proteins coming from four rust species (*Mli*, *Mlp*, *Pgt*, and *Pst*), and all contain at least one member from *Mli*. Tribes were grouped according to whether they contain members from *Mli* only or members from the two *Melampsora* species only or members from all four rust species. **(A)** Average tribe size is represented in colored bars, with species of origin of tribe members shown. The total number of tribes and *Mli* members in each group is represented with dots and white font, respectively. **(B)** For each group, the average fraction per tribe of proteins with a PFAM annotation is shown with standard error bars.

In total, these selected 200 tribes contained 2642 proteins with representatives from all four rusts, including 725 proteins from the flax rust fungus. Of the 200 tribes, 105 (52.5%) had members from all four rust species, 75 (37.5%) had members from *Mli* and *Mlp* only and 16 (8%) were unique to *Mli* (Figure 5A). Out of 725 *Mli* predicted effectors, we found only 34 (4.6%) that were in tribes specific to *Mli*, whereas 235 (32.4%) had close homologs only in *Mlp*, and 451 (62.2%) had close homologs across the four rust species. Hence, it seems that the majority of the top-ranking candidate effectors are conserved across the four rust fungi studied here. Tribes containing members from all four rust fungi were relatively large, with an average of 18.2 proteins per tribe and a similar number of members from each species. This indicates that, across the 105 rust fungi-conserved tribes, there is no major shift toward expansion or reduction in gene numbers, although these may be observed at the individual tribe level. Indeed, 5 tribes out of 200 had one member from *Mli* and two members from either *Mlp*, *Pst*, or *Pgt*. These could potentially represent deletions occurring within one species, proteins missing from the annotation or expansion of gene families across several rust fungi genera. Overall, out of the 2642 proteins present in the 200 tribes, 1706 (64.5%) had a predicted SP. Out of the 725 *Mli* proteins in the 200 tribes, there were 395 (54.5%) with a predicted SP, representing 36% of the predicted secretome. Several of these tribes, particularly the larger ones, contain some members with a predicted SP and others lacking an SP; all are considered candidate effectors in this study. For example, tribes 54 and 55 (65 members each) consist of putative aspartate proteases and carboxipeptidases, respectively (annotation available for 63 proteins out of 65, for both tribes). However, only 27 and 29 proteins in each tribe, respectively, have an annotated SP. These tribes may include some members that act on intracellular substrates and others with extracellular activity. Such is the case for fungal chitinases which can function both intra- and extra-cellularly (Duo-Chuan, 2006). In our set, chitinases corresponded to tribe 58 with 32 out of 61 members with an annotated SP. This suggests that some effectors may have evolved from their non-secreted ancestors through the addition of an SP, with a role to perform the same or a similar biological function outside of the fungal cell, e.g., on the host cell wall or inside the host cell.

#### Shared candidate effectors enriched in predicted apoplastic enzymes

We found that PFAM functional annotation was primarily obtained for tribes that consisted of members from all four rust fungi (Figure 5B). The most reliable annotations, i.e., the lowest *E*-values in *BlastP*, were for catalytic enzymes such as glycoside hydrolases and proteases. We found that rust fungi share effectors that are likely involved in (1) degradation of the host physical barriers to infection, (2) inhibition of host immunity and (3) nutrient acquisition. Among the secreted glycoside hydrolase families (GH), we found putative cell wall degrading enzymes including cellulases (GH3, 5, 7, 10, 12, 17, 61), callases (GH16), mannanases (tribe 193, GH76), xylanases (GH10), pectinesterases (tribe 190, Hesp-412), and cutinases (tribe 94). Additionally, we identified a number of candidate effectors that may act in detoxifying the environment or inhibiting immune response signaling, including secreted superoxide dismutases (tribes 91 and 368) and thioredoxins (tribe 620). Other candidate effectors were predicted to be catalytic enzymes that target sugars and proteins either to suppress host immunity or to derive nutrients. Among these, we found sugar degrading enzymes (GH27, 31) and a number of predicted secreted proteases including subtilases (tribe 293), serine carboxipeptidases (tribe 55) and aspartate proteases (tribe 54). From their predicted functions, several of these candidate effectors would likely operate on substrates in the host apoplast.

#### Identification of putative translocated effectors

Previous work has shown that all four characterized Avr proteins from *Mli* are expressed in haustoria and translocated to the host cell where they are recognized by cytoplasmic TIR-NB-LRR resistance proteins. We found that all four *Mli* Avr proteins were in tribes either specific to *Mli* or in tribes shared only within the *Melampsora* genus. The tribes containing AvrP4 and AvrP123 were specific to *Mli*. Previously, it was found that AvrP4 is present broadly across the *Melampsora* genus and shows signatures of positive selection, at several coding positions, resulting in extensive diversity at the protein level (Van Der Merwe et al., 2009). Most similarity among homologs from *Mli* and *Mlp* resides in the N-terminus end of the proteins, which contains the SP domain, whereas the C-terminal end shows more polymorphism and the signature of positive selection. Here, the closest AvrP4 homologs from *Mlp* were in a separate tribe specific to *Mlp*, perhaps reflecting the fact that the clustering into tribes was performed with predicted mature proteins. In contrast, AvrL567 and AvrM were in tribes shared between *Mli* and *Mlp* and these tribes had no proteins from *Pgt* or *Pst*. These four Avr protein families may have evolved after the divergence of *Melampsora* and *Puccinia.* Also, the average sizes of tribes specific to *Mli* (2.1 proteins) or the two *Melampsora* species (8.9 proteins) were smaller than that of tribes shared across the *Melampsora* and *Puccinia* species studied here (18.2 members). This suggests that relatively small, *Melampsora*-specific tribes may be enriched in translocated effectors.

Other candidate effectors that are putatively translocated to the host cell included rust fungal homologs of the translocated chorismate mutase Cmu1 effector from *U. maydis* (tribe 558) and of the bean rust pathogen RTP1p effector (tribe 176, hesp-327), which translocates to host cells to function as a putative protease inhibitor (Kemen et al., 2005; Pretsch et al., 2013). These two known effectors, with homologs in all four rust fungi studied here, do not have known avirulence properties. Interestingly, several additional candidate effectors expressed in haustoria (e.g., tribe 26- hesp-642, tribe 52-hesp-c46 and hesp-c9, tribe 304-hesp-1266, tribe 77-hesp-735) corresponded to predicted secreted proteins with a putative nuclear localization signal and PFAM domains similar to those found in nuclear proteins. Further work will be required to assess whether they are translocated to the host cell, as suggested by their PFAM annotation.

## Discussion

Here, we report the annotated genome sequence of *Melampsora lini*, providing a genomic resource on a well-established pathogen for research into rust diseases. To our knowledge, it represents the largest fungal genome sequenced so far. Interestingly, genomes from *Melampsora* species can be significantly different in size, with genomes of the poplar and flax rust pathogens estimated at ~100 and ~220 Mbp, respectively. It is unclear what mechanisms generate such variation, although a simple genome duplication is unlikely as we have found a comparable number of genes between *Mli* and *Mlp* (16,271 and 16,399 respectively; Duplessis et al., 2011a). The comparatively large genome size of *Mli* can be explained in part by the presence of a greater amount of interspersed repeats (mostly related to transposable elements), as repeats represent ~45% of both *Mli* and *Mlp* genomes, but with a *Mli* genome more than twice the size of the *Mlp* genome. Similarly, there appears to be a higher absolute amount of non-repetitive sequences in *Mli* than in *Mlp*, explaining further the size difference between the two related genomes. There was significant agreement between gene models of *Mli* and *Mlp* or *Pgt*. The RNAseq data collected from infected leaf tissue 6 days post infection served to support gene predictions for *Mli* genes expressed during infection in hyphae and haustoria. Additionally, by aligning *Mlp* and *Pgt* proteomes to *Mli* genome, we were able to annotate genes that may be missing from our infection transcriptome, because they are expressed at other life-stages than captured using the RNAseq data from infected flax leaves, providing they had homologs from *Mlp* or *Pgt*. Thus, we are confident that our assembled sequence and annotation cover extensively the gene space of *M. lini*.

We have sequenced the hybrid isolate CH5, which carries the four characterized Avr proteins and eight genetically identified but not yet cloned avirulence genes. The candidate effectors reported in this study provide a starting point for screening for the corresponding avirulence phenotypes of these unknown Avr proteins. With respect to assembling a consensus haploid genome of *Mli*, using this isolate posed a number of challenges and resulted in a significant amount of genome fragmentation. The polymorphism between the alleles inherited from the parental isolates C and H generated numerous conflicting assemblies that could not be resolved by the assembly software. This may be true particularly at some effector loci where two significantly diverged alleles exist in CH5 or where the effectors occur in large multigene families surrounded by repeats. For example, at the *AvrL567* locus, the virulence allele contains only one gene (*AvrL567-C*) whereas the avirulence allele carries a tandem gene duplication (*AvrL567-A* and *-B*; Dodds et al., 2004). Reads from the three genes were assembled to form a single haploid consensus ORF. Although the predicted protein had “correct” sequence that allowed effector prediction, the resulting assembly does not reflect the complexity at this locus, i.e., unequal number of genes between the two alleles. Similarly, assembling the regions containing *AvrM* presented multiple challenges due to the presence of repeats in the flanking regions and the similarity in the ORF of the *AvrM* paralogs (Catanzariti et al., 2006). This resulted in a single scaffold being assembled for the coding space from all paralogs as well as separate scaffolds for the flanking regions. The “collapsed” scaffold was missing the first 10 nucleotides in the ORF, resulting in an incorrect protein prediction. Overall, such issues are typical for assemblies of polymorphic diploid genomes (Yandell and Ence, 2012) and are expected to occur particularly in regions containing complex gene families. However, based on the observation that most (99.8%) filtered haustorial ESTs had strong matches in the assembly, we expect that at least one member of each complex effector gene family is present in the assembly. Further progress can still be made to reduce the fragmentation of the assembled genome of *Mli* and improve gene calls. For example, the F2 segregating population derived from CH5 can be exploited to place the genomic scaffolds on a genetic map. An initial attempt to estimate the synteny between *Mli* scaffolds and the comparatively large *Mlp* scaffolds proved computationally too difficult due to the high level of fragmentation of the *Mli* genome, although it may become feasible with improved assemblies. Here, we have used RNAseq transcripts aligned to the genome for calling genes; nonetheless, a subset of *de novo* assembled RNAseq transcripts that failed to align to the genome may be helpful to identify missing gene calls typically due to genome mis-assemblies or gaps (Haas et al., 2003). In the future, particular efforts will focus on curating manually the genome annotation in regions of interest with a specific focus on haustorially-expressed genes encoding secreted proteins, similar to the approach taken by Duplessis et al. (2011a).

We have identified a large number of candidate effectors in *Mli* that show variable degrees of evolutionary conservation, i.e., shared across the four rust species included in this comparison, shared between the two *Melamspora* species or specific to flax rust. Within the 200 selected tribes out of 940 described here, we found 37% of *Mli* candidate effectors to be specific to the *Melampsora* genus, which contrasts with the ~74% of all small secreted proteins present in *Mlp* but without *Blast* matches in *Pgt* (Duplessis et al., 2011a). The difference may result from our clustering and tribe-ranking approaches, which emphasized larger (more shared) tribes and does not solely rely on secretome predictions. Here, we have highlighted candidate effectors that may contribute to plant pathogenicity across rust fungi, largely recapitulating previous related studies (Duplessis et al., 2011a; Saunders et al., 2012; Garnica et al., 2013), as well as potential determinants of host specificity in *Mli*. Importantly, all known *Mli* Avr proteins were in tribes that are either specific to *Mli* alone or *Mli* and *Mlp*, whereas a majority of the conserved rust fungal tribes contain enzymes with expected apoplastic activity. In flax, all resistance genes identified so far are predicted to act in the cytoplasm (Ellis et al., 2007). Taken together, this may indicate that rust fungal tribes specific to the genus level are enriched in intracellular effectors and thus may be a primary source of Avr proteins targeted by host intracellular immune receptors. It is unclear whether these genus-specific effectors determine host specificity of rust fungi species via their virulence action, and not just their potential avirulence properties. So far, most of the research on rust fungal effectors has focused on these translocated effectors (see review by Duplessis et al., 2011c). In contrast, little is known of the putative apoplastic effectors of rust fungi identified here and in previous studies (Duplessis et al., 2011a; Hacquard et al., 2012; Saunders et al., 2012; Garnica et al., 2013). The apparent wide conservation across rust fungi of some of these apoplastic effectors that perform a more general virulence function on a wide variety of hosts (e.g., cellulases) makes them particularly interesting for future studies. In the plant-pathogenic Ascomycete fungus *Cladosporium fulvum*, several apoplastic effectors have been characterized, including some that are recognized extra-cellularly by immune receptors (reviewed in Wit et al., 2009). For example, *Ecp6* and Avr4 function as chitin-binding proteins that inhibit host chitin-elicited immunity and a host-chitinase inhibitor, respectively (Van Den Burg et al., 2006; De Jonge et al., 2010). Further work is required to assess whether candidate apoplastic effectors from rust fungi have similar roles to the *C. fulvum* effectors and whether there are components of host immunity that may target them in the apoplast.

To fine-tune the search for the effector complement of the flax rust fungus, we took advantage of previous knowledge of *Mli* Avr proteins and other known rust effectors. Tribes of candidate effectors were prioritized for future studies, including functional characterization, if, similar to the previously known rust effectors, they ranked among the selected top 200. The similarity-based clustering of proteins into tribes used here is beneficial for identifying conserved gene families although just as any sequence-based clustering approach, its power decreases when dealing with related genes under accelerated rates of evolution, such as Avr gene families. Regarding the ranking approach, we elected to give weight to the presence of known effector motifs, although previous work has found that no obvious protein motif broadly characterized effectors from rust fungi species (Saunders et al., 2012). Also, effectors with no identified homologs in the flax rust fungus or another rust fungus studied here would appear in tribes of size 1, and would likely be ranked low, despite their biological relevance. Thus, it should be noted that tribes with low or intermediate ranking may still correspond to effectors, e.g., tribe 400 ranks 371 out of 940 and consists of putative extracellular invertases, probably essential to degrade sugars outside of the fungal cell. Also, a small number of effectors may still be missing from our predictions. This could result from difficulties in generating the assembly for some of them or missing gene calls, although coverage of ESTs from haustoria and CEGMA analyses suggests this is a limited occurrence (~5%). In addition, our filtering criteria, while enriching for likely effectors could generate a number of false negatives, e.g., in the case of effectors without a conventional eukaryotic secretion signal, such as the barley powdery mildew effectors AVR_*k*1_ and AVR_*a*10_ (Ridout et al., 2006). Likewise, the 50 amino acid cutoff for gene prediction does not allow discovery of very small effectors, such as the bean rust candidate effectors PIG11 and PIG13 (24 and 31 amino acids respectively; Hahn and Mendgen, 1997). Also, a mis-annotated 5′ end or a real SP that falls just under the cut-off for prediction could cause some effectors to be missing from the effector complement. However, we limited these problems by forming tribes that contained even just one predicted secreted member and enriching those that were expressed in haustoria. Generally, however, based on our use of known Avr proteins and rust effectors to help set the parameters of the pipelines for genome assembly and annotation and effector prediction, we are confident that the bulk of *Mli* effectors are contained in our set.

Our findings agree with trends previously reported for non-rust biotrophic plant pathogens. Specifically, our results support the notion that evolution of obligate biotrophy is associated with the loss of some metabolic pathways (Kemen et al., 2011), although our results illustrate that the degree to which pathways can be affected may vary. For example, an almost complete pathway for sulfate metabolism was identified in *Mli*, and previously in *Mlp*, but appears to be absent in *Puccinia* (Duplessis et al., 2011a; Garnica et al., 2013), Ascomycetes such as the barley powdery mildew pathogen *Blumeria graminis* f.sp. *hordei* (Spanu et al., 2010) and even Oomycetes such as *Hyaloperonospora arabidopsidis* the downy mildew pathogen of *Arabidopsis thaliana* (Baxter et al., 2010). Consistent with findings on other rust fungi (Duplessis et al., 2011a; Garnica et al., 2013), in *Mli* the probable loss of the ability to import and metabolize nitrate or nitrite appears to be coupled with an expansion in the number of amino-acid and oligopeptide transporters, compared to non-biotrophic basidiomycetes, which would allow accumulation of host-derived organic nitrogen sources. During infection by *Mlp*, transporter proteins are mostly expressed after haustorial formation (~48 h post infection; Duplessis et al., 2011b), supporting the view that they are involved in the uptake of host-derived nutrients and possibly also the efflux of virulence factors and influx of plant anti-fungal compounds for detoxification. Assigning the direction of transport and the nature of the cargo translocated by the numerous transporters described here will require significant further investigation.

Finally, studies on *M. lini* collected from wild populations infecting the native Australian wild flax (*Linum marginale*) has revealed the existence of two lineages of *Mli*, namely the AA and AB lineages, where A and B refer to the genetic constitution of the two haploid nuclei in the dikaryon (Barrett et al., 2007). The lineages exhibit substantial differences in terms of virulence and life-style, with lineage AA capable of both sexual and asexual (clonal) reproduction and lineage AB only found to reproduce clonally (Nemri et al., 2012). The complete life-cycle of *Mli* contains five different spore stages, all occurring on flax (Lawrence et al., 2007; Ravensdale et al., 2010). In this study, we were interested in genes expressed during infection with uredospores, the asexual spore stage, of isolate CH5 (lineage AA). In the future, it will be interesting to compare it with the infection transcriptome following inoculation with the four spore stages forming the sexual cycle. Also, comparing the genomes and transcriptomes of isolates of lineage AA and AB may give insight into how much within-species diversity can be found in candidate effectors or candidate genes mediating environmental adaptation and life-history differences.

In conclusion, we have identified a large number of candidate proteins potentially involved in multiple aspects of infection of flax by *Mli* uredospores. These aspects include: (1) penetration of host tissue and colonization, with cuticle and cell wall degradation enzymes; (2) detoxification and modification of host metabolism for suppression of host defenses and promotion of infection; and (3) hydrolysis and uptake of nutrients. Further work is needed to assign effector candidates and metabolic pathways to specific time-points of infection and specific fungal organs. In *Mli*, a technique for genetic transformation and gene silencing is available (Lawrence et al., 2010), creating the opportunity to dissect the role of candidate genes identified in this study, coming from *Mli* and other rust fungi. This provides a starting point for future investigations aiming to understand virulence in economically important rust fungi and developing innovative strategies to render crops resistant to them.

## Materials and methods

### Sample preparation

Genomic DNA was extracted from *Melampsora lini* reference isolate CH5 uredospores according to Justesen et al. (2002) with modifications. Approximately 100 mg of dried uredospores were ground with 1 g of acid washed sand using a pestle and mortar. The powder was transferred to a 15 ml polypropylene tube and resuspended in 2 ml of DNA extraction buffer (25 g/L D-sorbitol, 10 g/L sodium dodecyl sulfate; 8 g/L hexadecyltrimethylammonium bromide (CTAB), 10 g/L polyvinylpyrrolidone (PVP), 0.8 M NaCl, 20 mM EDTA pH 8.0, 0.1 M Tris HCl pH 8.0). Five microliters of 100 mg/ml RNaseA were added and the samples were incubated at 65°C for 30 min. Ten microliters of 20 mg/ml proteinase K were added and the samples were incubated at 65°C for a further 30 min before extraction using 3 ml of chloroform. DNA was precipitated by addition of 1 vol. isopropanol and DNA was recovered by centrifugation for 15 min at 16,000 g. DNA was washed with 75 % (v/v) ice-cold ethanol, air-dried and resuspended in 62.5 mM MOPS pH 7.0. DNA was then cleaned-up using a Qiagen G/20 genomic-tip according to the manufacturer's instructions. Four genomic DNAseq libraries were generated including one paired-end library of ~300 bp and three mate-pair libraries of sizes 2000, 3000, and 5000 bp (Table S1). All sequencing was performed at Macrogen Inc. (Seoul, Republic of Korea) and the Australian Genome Research Facility (AGRF, Sydney, Australia) using Illumina HiSeq2000 to produce reads of 100 bp. Additionally, an RNAseq library was generated from leaf material of host line Hoshangbad infected with isolate CH5 at 6 days post infection as in Catanzariti et al. (2006). In total, ~110 million raw 75 bp single-end reads were sequenced using Illumina Genome Analyzer II at AGRF. Finally, a haustorial-specific EST library of 2783 sequences was used, as described in Catanzariti et al. (2006), including 1961 ESTs not previously reported. The library was preprocessed with *Seqclean* (http://compbio.dfci.harvard.edu/tgi/software/) to remove polyA's and vector contamination (UniVec_Core library, http://www.ncbi.nlm.nih.gov/VecScreen/UniVec.html).

### *de novo* genome and transcriptome assembly

Prior to assembly, DNAseq and RNAseq reads underwent quality-based trimming using *Condetri* (Smeds and Kunstner, 2011) and trimming of Illumina adaptor sequence using *Trimmomatic* (Lohse et al., 2012). Removal of PCR duplicates from all four DNAseq libraries was done using f*ilterPCRdupl* (http://code.google.com/p/condetri) followed by removal of likely sequencing errors using *ErrorCorrectio*n (Luo et al., 2012). The genome assembly and initial scaffolding were performed using *SOAPdenovo v2r215* (Luo et al., 2012). After testing k-mer values ranging from 37 to 47, a k-mer value of 41 was found to give the best results and was used to produce the assembly reported here. To close gaps in the scaffolded SOAPdenovo assembly, we used *GapCloser v1.12r1* (Luo et al., 2012) followed by scaffolding using *SSPACE v2.0* (Boetzer et al., 2011) for two rounds using the four paired DNAseq libraries. Only scaffolds longer than 200 bp were retained in the final genomic assembly. For the transcriptome analysis of infected leaf tissue, we obtained ~94 million reads from the ~110 million raw reads after quality-based filtering. We then filtered out RNAseq reads originating from flax by aligning the reads against the genome sequence of flax v1.0 (Wang et al., 2012) and a collection of flax ESTs (Fenart et al., 2010). Around 38% of the total RNAseq reads were removed as flax reads, leaving ~58 million reads (62% of the total), mostly from rust and potentially including some contaminant. Transcript assembly was done using two strategies. First, assembly-by-alignment to the genome sequence was performed using *Tophat*_*v2.0.6/Cufflinks*_*v2.0.2* (Trapnell et al., 2010). Second, genome-guided transcript assembly was done using *Trinity r2012-10-0*5 (Grabherr et al., 2011), coupled with *PASA r2012-06-25* to predict terminal exons (Haas et al., 2008).

### Gene prediction and annotation

*Ab initio* gene prediction was performed using: (1) *Augustus v2.5.5* (Stanke et al., 2006) with aligned ESTs as hints and *Ustilago maydis* as related species for training the gene finder; and (2) *GeneMark-es r2012-02-03* (Ter-Hovhannisyan et al., 2008). Spliced protein-to-genome alignment was performed using *Exonerate v2.2.0* (Slater and Birney, 2005) with Uniref90 (downloaded from www.uniprot.org) and complete proteomes of *Mlp* isolate 98AG31 (obtained from http://genome.jgi.doe.gov/) and *Pgt* isolate CDL-75-36-700-3 (race SCCL; obtained from http://www.broadinstitute.org/) (Duplessis et al., 2011a). *EvidenceModeler* (Haas et al., 2008) was used to combine ESTs, gene predictions, spliced protein alignments and transcript alignments. The annotation was then imported into *PASA* to update transcript predictions, add UTR's and alternative transcripts. *CEGMA* (Parra et al., 2009) was used to verify the quality of the assembly of the gene space in the genome and the annotation output by *EvidenceModeler*. *Blast2go* was used to filter out the predicted transposable elements in the final proteome set (Conesa et al., 2005). *Webapollo* was used for genome browsing and inspection of the annotation (Lee et al., 2013). *De novo* identification of repeats in the genome sequence was performed using *RepeatMasker* v4.0.1 (Smit et al., 1996).

### Rust effector prediction

An effector prediction pipeline modified from *PexFinder* (Torto et al., 2003) was set up to search the proteomes of all four rust fungi species, *Mli*, *Mlp*, *Pgt* (isolates specified above) and *Pst* isolate 130 (*Pst*; Cantu et al., 2011), obtained from D. Saunders, Sainsbury Laboratory, Norwich, UK. Proteins were selected if (1) they exceeded the cutoffs for SP prediction of 0.88 for S-probability in *SignalP v2.1-HMM* (Nielsen and Krogh, 1998) or 0.36 for D-score in *SignalP v4.1b* (Petersen et al., 2011); (2) the predicted cleavage site occured between amino-acid 10 and 40; (3) no transmembrane domain was predicted to occur after the cleavage site using *Tmhmm v2.0c*; and (4) the protein was not predicted to be mitochondrial by *TargetP v1.1* (Emanuelsson et al., 2007). Any protein from all four rust fungi proteomes that passed the selection criteria was subsequently used as a query in BlastP against the remainder of the proteomes and effector tribes were formed, as per Saunders et al. (2012). In order to group candidate effectors with functional and/or structural similarities in the effector domain, the clustering was performed using predicted mature proteins when a SP was detected. Real effectors appearing as false negatives in SP prediction, due to a mis-annotated 5′ end (as was the case for AvrM), or a correct SP with a prediction that falls under our cut-offs, were included in the clustering if one related protein had a predicted SP. Preventing the Markov clustering from being primarily driven by the SP resulted in (a) these “recovered” effectors being assigned to their correct tribe and (b) avoiding the formation of very large tribes potentially composed of effectors with greatly divergent or unrelated functional domains but with a conserved SP. This focus on the functional domain of effectors also meant that the evolutionary information contained in the SP domain was not used to form the tribes.

Aside from the number of members in a tribe, six features contributed to ranking the tribes. For each of these features, a score was calculated as per Saunders et al. (2012). This score is based on the number of proteins within a tribe that displayed a particular feature, relative to the likelihood of a tribe of the given size containing the same number of proteins with that particular feature by chance. Features assessed for each protein from a tribe included: [1] being a predicted secreted protein, [2] having a BLAST match against HESPs (Catanzariti et al., 2006), or [3] haustorial ESTs and [4] being a small cysteine-rich protein. These four features were given high weight in the formula described below. Additionally, features included having [5] one or more effector motifs such as [L/I]xAR, [R/K]CxxCx12H, RxLR, [Y/F/W]xC, YxSL[R/K], or G[I/F/Y][A/L/S/T]R between amino acids 10–110, or a nuclear localization signal identified using *NLStradamus* (Nguyen Ba et al., 2009), or [6] one or more internal repeats identified using *T-reks* (Jorda and Kajava, 2009). These two features were assigned minor weight in the formula below. To emphasize shared properties among members of a tribe rather than particular features of one member, each feature was scored as a single 0 or 1, e.g., having two [Y/F/W]xC motifs and a nuclear localization motif was treated the same as having just one [Y/F/W]xC motif. Weights for all six features were combined to produce an overall score used to rank the tribe, calculated as = *log*_2_ ([1] + [2] + [3] + [4]) × (1 + 0.1 × ([5] + [6])). Manual curation of the ranked list was performed to remove tribes with less than 10% of secreted members. Out of the top-ranking 232 tribes, 32 tribes were removed (gray lines in Additional file 2, “Ranked tribes incl. *Mli* members”) giving a list of the top selected 200 tribes described here. These top 200 tribes and their overall and individual feature scores were visualized using *Circos* (Krzywinski et al., 2009). The proportion of proteins with a PFAM score was also assessed, but did not contribute to tribe ranking, as many fungal and oomycete Avr proteins do not have recognizable PFAM domains. PFAM categories based uniquely on domain-recognition without associated function were removed, including cysteine-rich secretory protein. A cut-off of E-5 was used.

## Author contributions

Adnane Nemri, Claire Anderson, Diane G. O. Saunders, Narayana M. Upadhyaya, and Joe Win performed computational analyses. Peter N. Dodds, Jeffrey G. Ellis, David A. Jones, and Sophien Kamoun designed and supervised the research. All authors contributed to manuscript writing.

### Conflict of interest statement

The authors declare that the research was conducted in the absence of any commercial or financial relationships that could be construed as a potential conflict of interest.

## Supplementary material

Table S1 Libraries used for the assembly of the genome and infection transcriptome of the flax rust pathogen *Melampsora lini* isolate CH5.

Additional file 1 (.zip) Fasta files of *M. lini* genomic scaffolds and proteome including Blast2go annotations as sequence descriptors where available.

Additional file 2 (.xlsx) Microsoft Excel workbook containing tables with [1] Complete list of tribes with members from four rust species, [2] *in silico* characterization of the 940 tribes of candidate effectors containing at least one member from *Melampsora lini* (gray lines correspond to tribes removed from the top 200), [3] top 200 *Melampsora lini* tribes with PFAM information where available and [4] transporters found in *Melampsora lini* genome with putative substrate where available.
