# Supplementary material for: The genome sequence and effector complement of the flax rust pathogen Melampsora lini
Source: Front Plant Sci. 2014 Mar 24;5:98. doi: 10.3389/fpls.2014.00098 (PMC3970004; doi:10.3389/fpls.2014.00098)
Supplement: Table S1 — Libraries used for the assembly of the genome and infection transcriptome of the flax rust pathogen Melampsora lini isolate CH5. [file DataSheet2.PDF]

Table S1. Libraries used for the assembly of the genome and infection transcriptome of the flax rust pathogen *Melampsora lini* isolate CH5.

Library type and tissue	sequencing service provider	type	average insert size (bp)	read count	base coverage depth
DNAseq from spores	AGRF	paired-end	295 ± 57	2x101M	92x
	AGRF	mate-paired	2000 ± 500	2x60M	54x
	Macrogen Inc.	mate-paired	3056 ± 331	2x43M	40x
	Macrogen Inc.	mate-paired	~5000	2x33M	30x
	pooled	unpaired	101	64M	29x
RNAseq from infected plant tissue	AGRF	unpaired	75		

During read pre-processing of the four DNAseq libraries, some reads lost their mates and became unpaired. Those reads were pooled to make the 'pooled' library of unpaired reads.
